# Arbitrary Ca^2+^ regulation for endothelial nitric oxide, NFAT and NF-κB activities by an optogenetic approach

**DOI:** 10.3389/fphar.2022.1076116

**Published:** 2023-01-10

**Authors:** Tomoyasu Yamanaka, Takatoshi Ueki, Mitsuhito Mase, Koichi Inoue

**Affiliations:** ^1^ Department of Neurosurgery, Nagoya City University Graduate School of Medical Sciences, Nagoya, Japan; ^2^ Department of Integrative Anatomy, Nagoya City University Graduate School of Medical Sciences, Nagoya, Japan

**Keywords:** optogenetics, vascular endothelial cells, calcium, nitric oxide, microarray, inflammation

## Abstract

Modern western dietary habits and low physical activity cause metabolic abnormalities and abnormally elevated levels of metabolites such as low-density lipoprotein, which can lead to immune cell activation, and inflammatory reactions, and atherosclerosis. Appropriate stimulation of vascular endothelial cells can confer protective responses against inflammatory reactions and atherosclerotic conditions. This study aims to determine whether a designed optogenetic approach is capable of affecting functional changes in vascular endothelial cells and to evaluate its potential for therapeutic regulation of vascular inflammatory responses *in vitro*. We employed a genetically engineered, blue light-activated Ca^2+^ channel switch molecule that utilizes an endogenous store-operated calcium entry system and induces intracellular Ca^2+^ influx through blue light irradiation and observed an increase in intracellular Ca^2+^ in vascular endothelial cells. Ca^2+^-dependent activation of the nuclear factor of activated T cells and nitric oxide production were also detected. Microarray analysis of Ca^2+^-induced changes in vascular endothelial cells explored several genes involved in cellular contractility and inflammatory responses. Indeed, there was an increase in the gene expression of molecules related to anti-inflammatory and vasorelaxant effects. Thus, a combination of human blue light-activated Ca^2+^ channel switch 2 (hBACCS2) and blue light possibly attenuates TNFα-induced inflammatory NF-κB activity. We propose that extrinsic cellular Ca^2+^ regulation could be a novel approach against vascular inflammation.

## 1 Introduction

Vascular endothelial cells form the vascular endothelium, a monolayer of cells lining the lumen of blood vessels, and contribute to blood circulation throughout the body by preventing blockages through anticoagulation. In addition to supporting blood circulation, vascular endothelial cells also release many bioactive molecules that regulate vasoconstriction and dilation (Bauer and Sotnikova, 2010; Zhao et al., 2015; Kruger-Genge et al., 2019). One of these molecules is nitric oxide (NO), a small gaseous molecule that is produced in vascular endothelial cells and then diffuses to and permeates the cell membranes of surrounding cells. Activation of soluble guanylate cyclase by NO in vascular smooth muscle leads to an increase in cyclic guanosine 3′,5′-cyclic monophosphate (cGMP). cGMP then promotes actin-myosin chain relaxation by decreasing Ca^2+^ influx in vascular smooth muscle cells, thus maintaining vascular function (Bauer and Sotnikova, 2010; Zhao et al., 2015; Kruger-Genge et al., 2019).

NO is usually synthesized in vascular endothelial cells by endothelial NO synthase (eNOS). eNOS is constitutively present in vascular endothelial cells, and its activity is regulated by the intracellular Ca^2+^-calmodulin system (Forstermann et al., 1991; Pollock et al., 1991). In this system, specific stimulation of vascular endothelial cells leads to Ca^2+^ influx, resulting in the formation of the Ca^2+^-calmodulin complex that then binds to eNOS, followed by migration of the complex into the cellular caveola, which promotes NO production. Additionally, phosphorylation of eNOS also modulates its activity. Alternatively, some stimuli result in increased eNOS protein expression levels, which facilitates increased NO production (Inoue and Xiong, 2009; Bauer and Sotnikova, 2010; Rafikov et al., 2011). The mechanisms involved in NO production hint at the clinical importance of NO. When vascular endothelial dysfunction occurs in the early stages of cardiac ischemia, NO production can become insufficient. Reflecting this, during angina pectoris or myocardial infarction, treatment with acute sublingual administration of nitroglycerin increases blood NO levels, possibly resulting in coronary vasodilation and enhanced cardiac blood supply. In addition, the test for coronary artery spasm involves cardiac catheterization-administered acetylcholine, which induces NO production, predominantly in the functional vascular endothelial cells over vascular smooth muscle cells, resulting in vascular dilation. On the other hand, acetylcholine-induced production of NO is not sufficient in dysfunctional endothelial cells; therefore, acetylcholine-induced contractions occur predominantly in vascular smooth muscle cells, leading to vasospasm (Ong et al., 2012; Ong et al., 2014). Thus, decreased NO production due to endothelial dysfunction minimizes vasodilatory capacity, resulting in arterial stiffness and a tendency for thrombosis development, which may further contribute to development of diseases, such as cerebral infarction, myocardial infarction, and peripheral arterial occlusion (Daiber et al., 2019).

In addition to NO production, an increase in intracellular Ca^2+^ in vascular endothelial cells facilitates prostaglandin production, which acts on vascular smooth muscle cells to induce vasodilation and binds to its receptors on platelets to activate adenylate cyclase, inhibiting platelet activation and promoting anti-thrombotic effects (Weksler et al., 1978; Braune et al., 2020). Elevation of Ca^2+^ in vascular endothelial cells also plays important roles in the immune response, including contributing to the regulation of E-selectin and VCAM-1 gene expression, or regulating immune cell adhesion and tissue infiltration, which is implicated in the development of atherosclerosis (Kielbassa-Schnepp et al., 2001; Erdogan et al., 2007; Moccia et al., 2014).

To date, we have focused our studies of vascular function on molecules involved in Ca^2+^ dynamics and inflammation (Inoue et al., 2004; Inoue and Xiong, 2009; Sun et al., 2013; Zeng et al., 2015; Inoue et al., 2020) and have found that suppression of TRPM7, one of TRP family members, augments eNOS protein levels, resulting in increased NO production (Inoue and Xiong, 2009). Moreover, under high glucose conditions, TRPM7 is upregulated in vascular endothelial cells, resulting in decreased eNOS expression and NO production, and inhibition of TRPM7 restores these levels (Sun et al., 2013). Regardless of these adverse effects, TRPM7 plays a role in cardiovascular Mg^2+^ homeostasis, and inhibition of immune responses (Rios et al., 2020), which demonstrates the difficulty in targeting suppression of a single endogenous molecule as a therapeutic approach. As inhibition of TRPM7 enhances NO production, we have focused on other target molecules that regulate NO production and expression of related genes. If their activities can be controlled, extrinsic regulation of production of NO and other molecules may be possible, ultimately leading to the regulation of vascular tone and function. However, isolation of endogenous molecule-dependent targets could require extensive research on identification and validation of the target molecule, followed by discovery of specific interacting molecules through drug library screening, with further optimization of the molecular structure using *in silico* techniques (Hughes et al., 2011; Esch et al., 2015; Schneider, 2018). Indeed, because of the difficulties involved previously in developing therapeutic agents through this process, it may be useful to introduce exogenous molecules into vascular endothelial cells to actively modulate their functional activity, for example, by promoting Ca^2+^ influx.

Recently, a lot of research has been conducted in exploiting molecules that are not endogenously present in higher organisms, including human beings. Channelrhodopsin, an algal protein that acts as a cation channel, is one such molecule and is activated by specific wavelengths of light. Photoactivation of channelrhodopsin results in passive diffusion of ions according to their equilibrium potentials, leading to changes in membrane potential; in neuronal cells, this generates action potentials, often resulting in neuronal discharges. Targeting genes that confer channels such as channelrhodopsin has enabled external control of neuronal activity by light manipulation; this technique is known as optogenetics (Deisseroth, 2011; Deisseroth, 2015). Different types of optogenetic tools, including anion channels, have already been discovered and developed for biomedical usage, with one of the most common channels being channelrhodopsin-2 (ChR2). ChR2 is a non-specific cation channel, conducting multiple cations such as Na^+^ and Ca^2+^ (Lorenz-Fonfria and Heberle, 2014). Previously, whilst analyzing channel activity modified by redox status (Wu et al., 2017), we considered that Ca^2+^ influx controlled by means of an optogenetic strategy and consequent activation of the downstream pathways could stimulate NO production and other activities in vascular endothelial cells, facilitating beneficial vascular processes. Photoactivation of vascular endothelial cells using light sensors could dilate vessels and release hormones, which control blood flow, at certain regions of vasculature in the body. In this study, we attempt to employ a relatively new optogenetic tool to facilitate an increase in intracellular Ca^2+^ by blue light irradiation and provoke NO production and gene expression in vascular endothelial cells, to explore the possibility of regulating vascular inflammatory responses.

## 2 Materials and methods

### 2.1 Reagents and antibodies

The following reagents and antibodies were used: Fluo-8/AM (ABD21081, AAT Bioquest, Sunnyvale, CA, United States); DAF-FM/DA (SK1004-01, Goryo Kagaku, Sapporo, Japan); NucreoSpin RNA Plus (U0984B, Takara, Kusatsu, Japan); Lipofectamine 3000 (L3000001, Thermo Fisher, Waltham, MA, United States); O,O′-Bis(2-aminoethyl) ethylene-glycol-N,N,N′,N′-tetraacetic acid (EGTA; 348-01311, Dojindo, Kumamoto, Japan); N^G^-nitro-L-arginine methyl ester (L-NAME; 80210, Fuji Film, Tokyo, Japan); BAPTA/AM (T2845, TCI, Tokyo, Japan); protease inhibitor cocktail (S8820, Sigma-Aldrich, St. Louis, MO, United States); Dual-Luciferase Reporter System (E1910, Promega, Madison, WI, United States); mouse monoclonal antibodies against Flag (F1804, Sigma-Aldrich) and GAPDH (60004-1-Ig, Proteintech, Rosemont, IL, United States), rabbit monoclonal antibody against NF-κB p65 (#8242P, CST, Danvers, MA, United States), TNFα (HZ-1014, Proteintech).

### 2.2 Cell culture

HEK293 cells, a murine vascular endothelial cell line F2 (RCB 1994, Riken BRC, Tsukuba, Japan) (Toda et al., 1990) and a murine brain derived endothelial cell line b.End3 (CRL-2299, ATCC, Manassas, VA, United States) (Montesano et al., 1990) were grown in Dulbecco’s modified eagle medium with 10% fetal bovine serum and antibiotics.

### 2.3 Plasmid transfection and photostimulation

A plasmid expressing human blue light-activated Ca^2+^ channel switch 2 (hBACCS2) with bicistronic expression of mCherry was provided by Dr. Takao Nakata, Tokyo Medical and Dental University, Tokyo, Japan; (Ishii et al., 2015) through Addgene (Watertown, MA, United States).

A fragment of mCherry or hBAACCS2-IRES-mCherry was inserted into a pCAG-GS vector provided by Dr. Junichi Miyazaki, Osaka University, Osaka, Japan; (Niwa et al., 1991) with Flag-tag on hBACCS2 (pCAG-hBACCS2-mCherry). G-CaMP7 [provided by Dr. Junichi Nakai, Saitama University, Saitama, Japan through Riken BRC; (Ohkura et al., 2012)] was subcloned into the pCAG-GS vector.

For transfection, Lipofectamine 3000 (Thermo Fisher) was used according to the manufacturer’s instructions. Transfection efficiencies, as determined by mCherry-positive cells, were ∼50% in HEK293 and F2 cells, and ∼5% in b.End3 cells.

**FIGURE 1:**
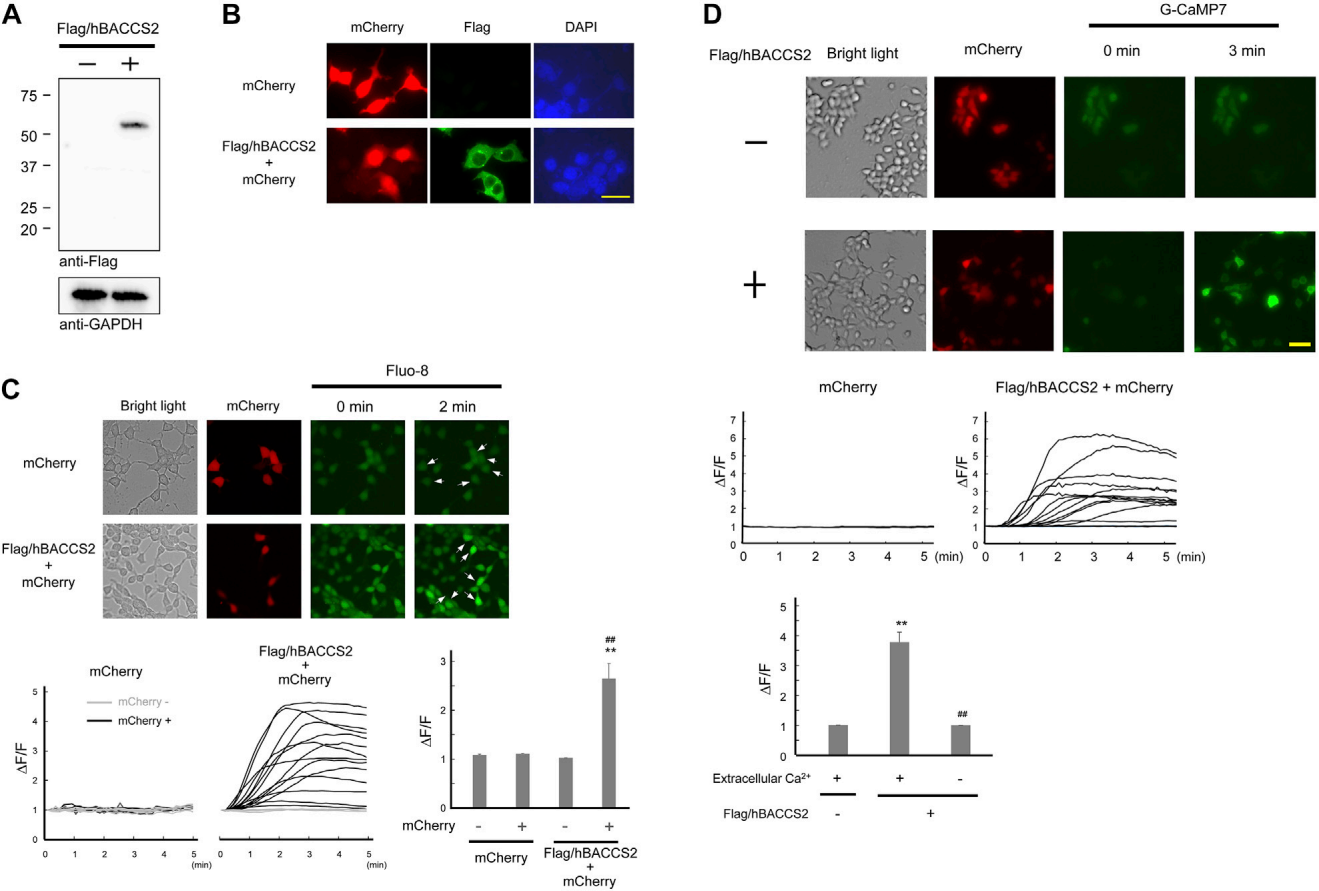
Flag/hBACCS2 leads to blue light-mediated Ca^2+^ increase in HEK293 cells. **(A,B)** Either Flag/hBACCS2-mCherry- or mCherry-expressing plasmid was transfected into HEK293 cells and incubated for 2 days. Cells were collected, followed by immunoblotting analysis **(A)** or fixed and Flag/hBACCS2 protein was visualized using an antibody to Flag **(B)**. The scale bar represents 20 μm. **(C)** Cells transfected with either Flag/hBACCS2-mCherry or mCherry were stained with Fluo-8/AM. Blue light was then applied (.2 Hz) and Fluo-8 signals were monitored. Arrows indicate mCherry-positive cells. Each trace represents fluorescence intensity from randomly selected cells. Bar graph shows the normalized fluorescence intensity of maximal values within 5 min in the different conditions as indicated. *n* = 11–24 from 3 independent experiments. **p < .01 vs. Flag/hBACCS2-negative cells, ^##^ *p* < .01 vs. mCherry-transfected cells, Student’s *t*-test. **(D)** Cells were transfected with G-CaMP7 and either Flag/hBACCS2-mCherry or mCherry, and photoactivated with blue light (.2 Hz). Each trace represents fluorescence intensity from randomly selected cells. Bar graph shows the normalized fluorescence intensity of maximal values within 5 min in the different conditions as indicated. The scale bar represents 50 μm *n* = 12–54 from 3 to 4 independent experiments. **p < .01 vs. Flag/hBACCS2-negative cells, ^##^ *p* < .01 vs. extracellular Ca^2+^-including condition, Student’s *t*-test.

**FIGURE 2:**
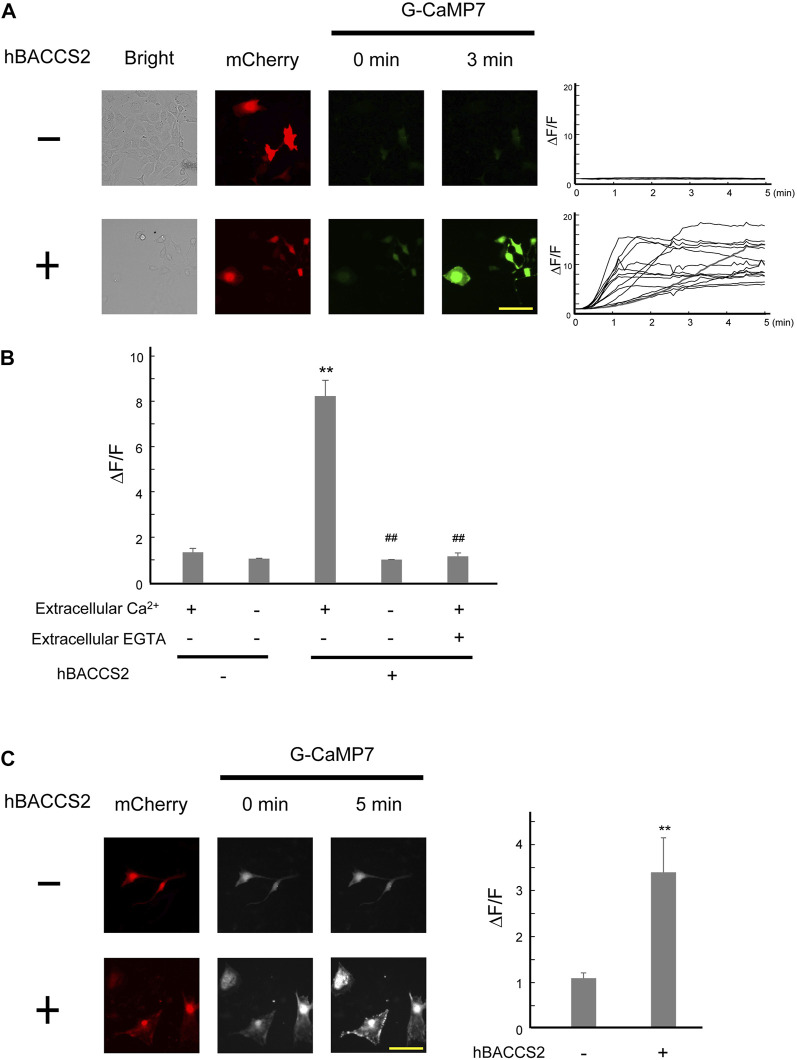
hBACCS2 leads to blue light-mediated Ca^2+^ increase in murine vascular endothelial cells. **(A)** Vascular endothelial F2 cells were transfected with G-CaMP7 and either hBACCS2-mCherry or mCherry, photoactivated with blue light (.2 Hz). Each trace represents fluorescence intensity from randomly selected cells. The scale bar represents 50 μm. **(B)** Bar graph shows the normalized fluorescence intensity of maximal values within 5 min in the different conditions as indicated. *n* = 13–58 from 3 to 6 independent experiments. **p < .01 vs. hBACCS2-negative cells, ^##^ *p* < .01 vs. extracellular Ca^2+^ +/EGTA - condition, one-way ANOVA with Fisher’s LSD post hoc test. **(C)** Brain microvascular b.End3 cells were transfected with G-CaMP7 and either hBACCS2-mCherry or mCherry and photoactivated with blue light (.2 Hz). Bar graph shows the normalized fluorescence intensity of maximal values within 5 min in the different conditions as indicated. The scale bar represents 100 μm *n* = 13–17 from 3 independent experiments. **p < .01 vs. hBACCS2-negative cells, Student’s *t*-test.

**FIGURE 3:**
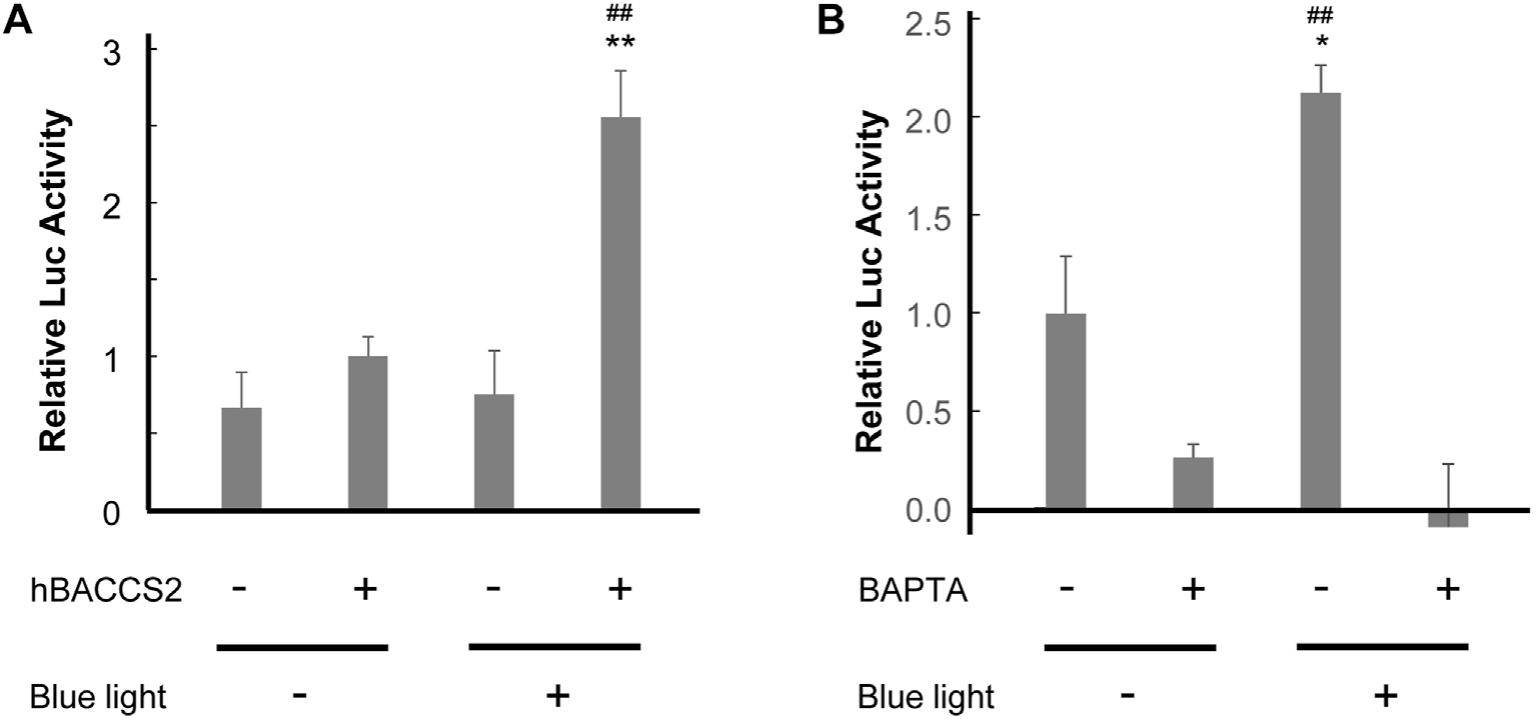
hBACCS2 leads to blue light-mediated NFAT activation in vascular endothelial cells. **(A)** F2 cells were transfected with pGL4.30[luc2P/NFAT-RE/Hygro], a firefly luciferase reporter plasmid containing an NFAT response element, pRL-TK, a control Renilla luciferase reporter plasmid, and either hBACCS2-mCherry or mCherry. Next day, 4 h after photoactivation (repletion of 2 min light irradiation and 18 min interval), the ratio of firefly luciferase activity to Renilla luciferase activity was calculated (*n* = 5). **p < .01 vs. hBACCS2 −, ^##^ *p* < .01 vs. blue light −, one-way ANOVA with Fisher’s LSD *post hoc* test. **(B)** F2 cells were transfected with pGL4.30[luc2P/NFAT-RE/Hygro], pRL-TK, and pCAG-hBACCS2-mCherry. On the following day after transfection, cells were pretreated with either 25 μM BAPTA/AM or vehicle for 30 min and photoactivation was then carried out for 4 h before being harvested. For each condition, the ratio of firefly luciferase activity to Renilla luciferase activity was calculated (*n* = 3). *p < .05 vs. blue light −, ^##^ *p* < .01 vs. BAPTA +, one-way ANOVA with Fisher’s LSD *post hoc* test.

**FIGURE 4:**
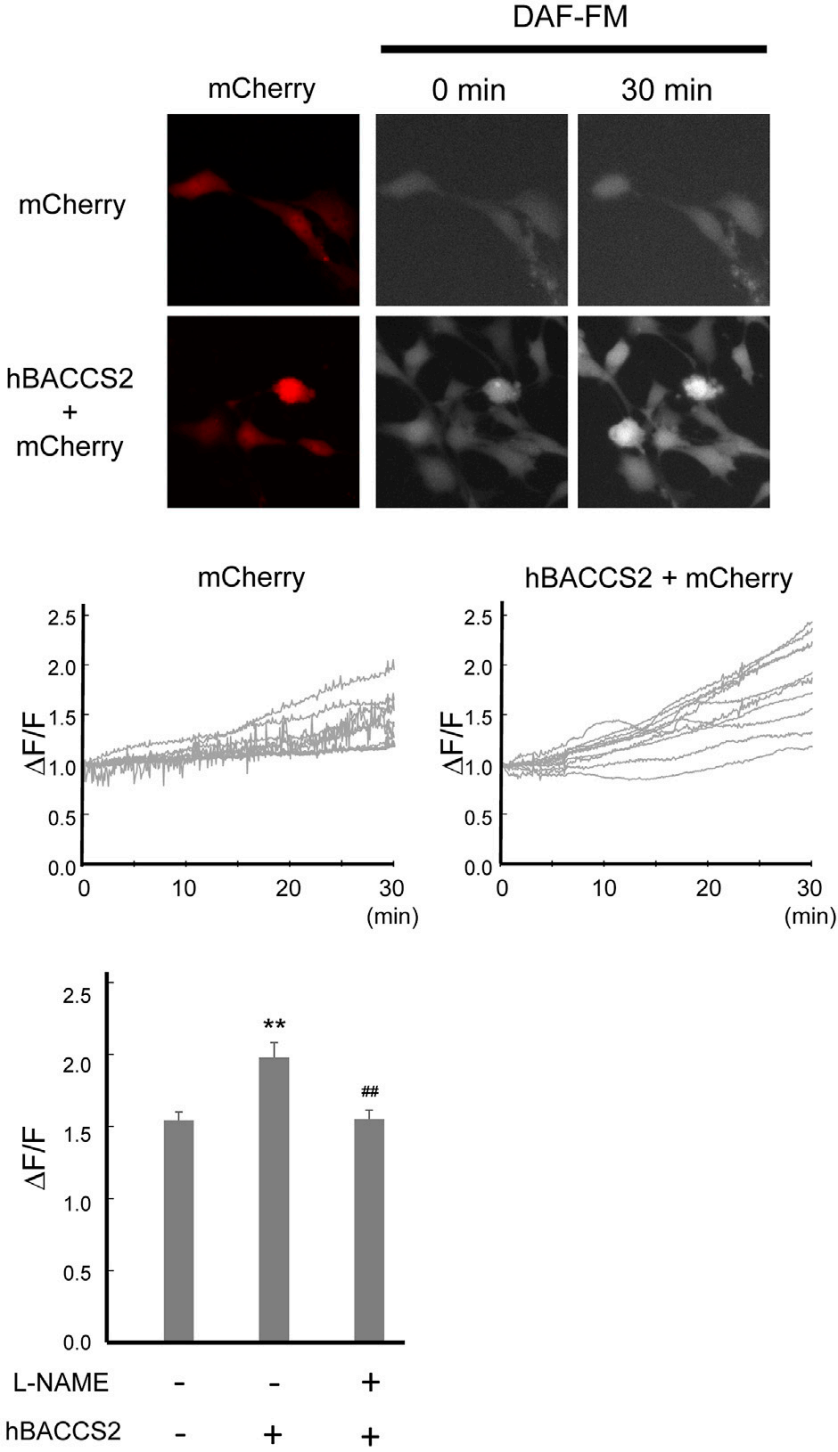
hBACCS2 leads to blue light-mediated NO production in vascular endothelial cells. F2 cells transfected with either hBACCS2-mCherry or mCherry were stained with DAF-FM/DA. Blue light was then applied (.2 Hz) and DAF-FM signals were monitored. Each trace represents fluorescence intensity from randomly selected mCherry-positive cells. Bar graph shows the normalized fluorescence intensity of maximal values within 30 min in the different conditions as indicated. *n* = 33–46 from 3–5 independent experiments. **p < .01 vs. hBACCS2-negative cells, ^##^ *p* < .01 vs. L-NAME - condition, one-way ANOVA with Fisher’s LSD *post hoc* test.

**FIGURE 5:**
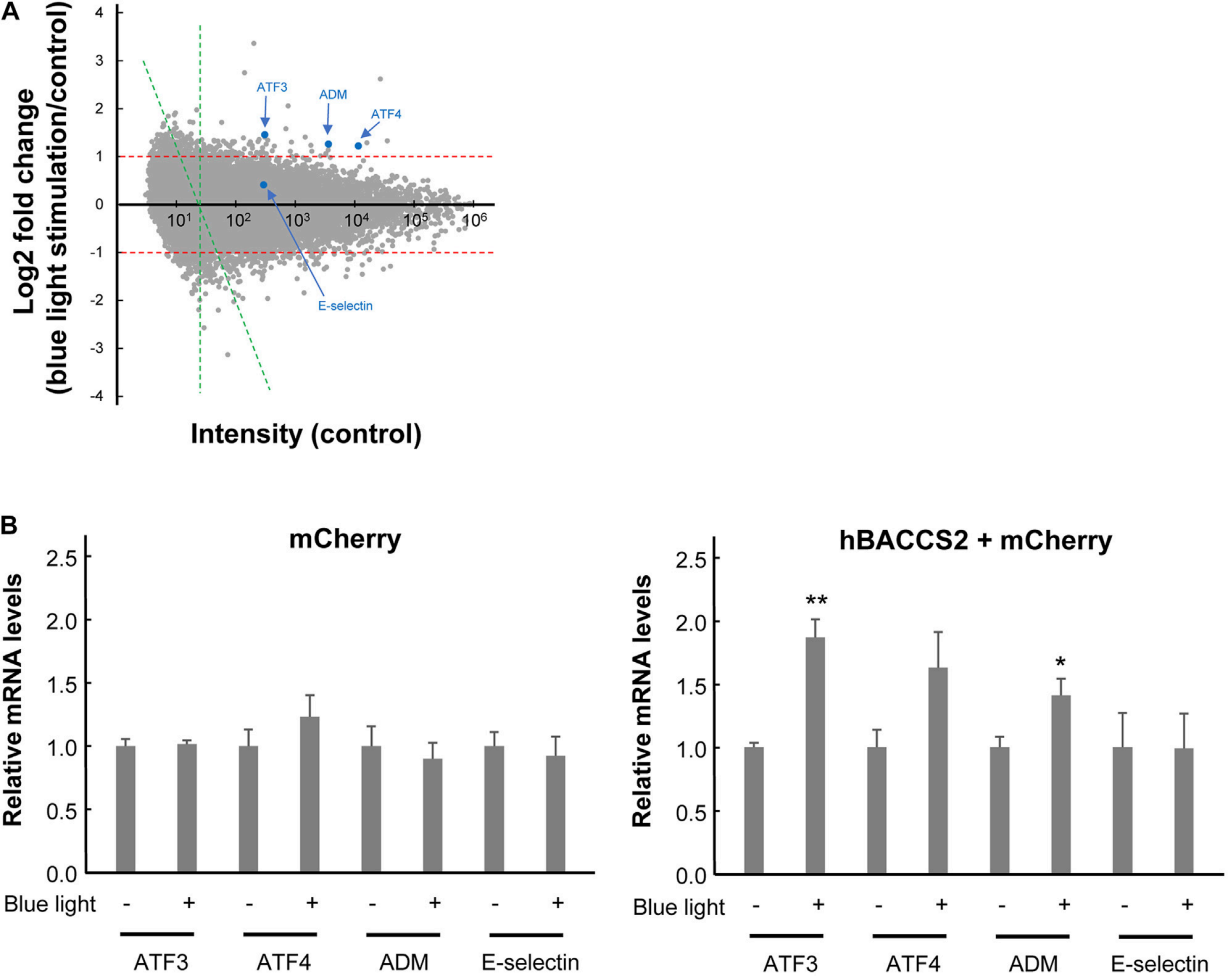
Gene expression profiling of hBACCS2-transfected F2 cells in the absence or presence of photoactivation. **(A)** Microarray analysis was performed comparing the gene expression profiles of hBACCS2-transfected F2 cells with or without blue light irradiation. A single dot is plotted for each gene. Some of representative genes were plotted with blue. Scatterplot comparing control intensities (x-axis) and their fold changes (log2-fold change, y-axis) was shown. A cut off intensity (x = 24) applied for pathway analysis (see methods) was indicated with green lines. **(B)** Quantitative real-time PCR was performed to validate mRNA levels of the indicated genes in F2 cells treated as described in **(A)**. *n* = 5–12. *p < .05, **p < .01 vs. blue light -, Student’s *t*-test.

**FIGURE 6:**
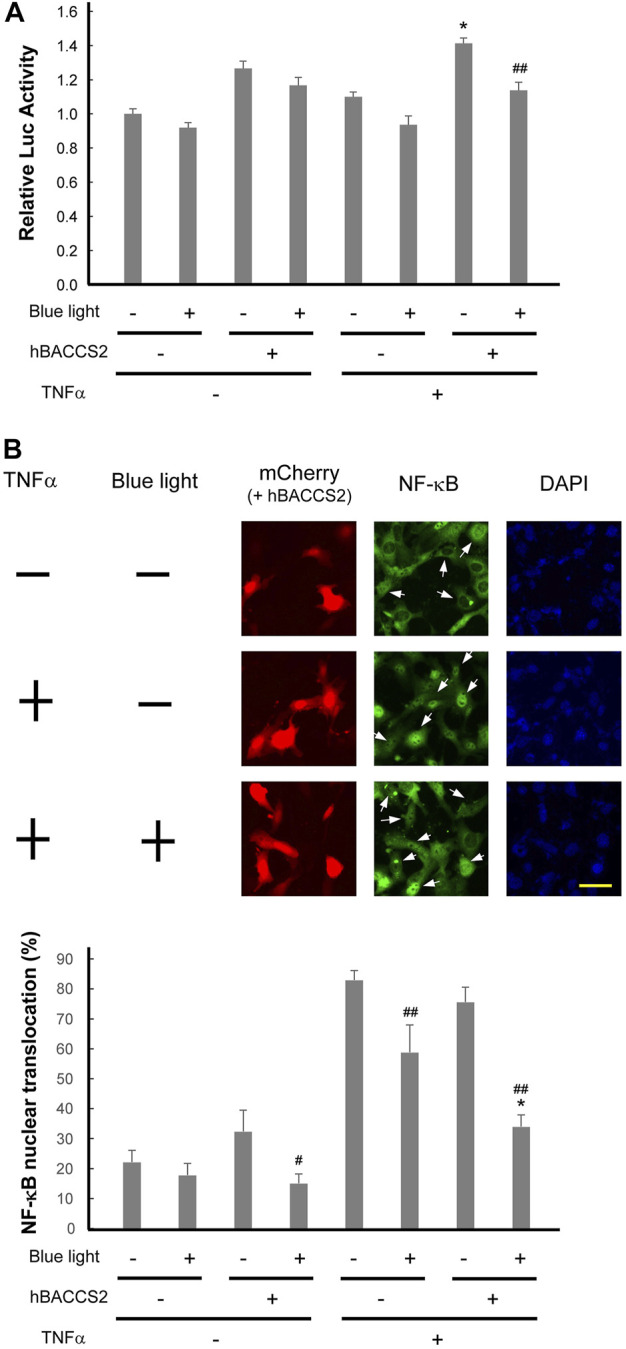
Photoactivation using hBACCS2 possibly attenuates TNFα-induced NF-κB activation in vascular endothelial cells. **(A)** F2 cells were transfected with pGL4.32[luc2P/NF-κB-RE/Hygro], a firefly luciferase reporter plasmid containing an NF-κB response element, pRL-TK, and either hBACCS2-mCherry or mCherry. Two h after photoactivation, they were either untreated or treated with 20 ng/ml TNFα for 2 h, followed by manipulation for reporter assay. (*n* = 6–7). TNFα-induced NF-κB activity was significantly attenuated with blue light irradiation in hBACCS2-transfected cells. *p < .05 vs. TNFα -, ^##^ *p* < .01 vs. blue light -, one-way ANOVA with Fisher’s LSD *post hoc* test. The effect of hBACCS2 was always significant (*p* < .01); this is not indicated in the panel. **(B)** F2 cells were transfected with either hBACCS2-mCherry or mCherry. In the absence or presence of 2 h after photoactivation, cells were either untreated or treated with 20 ng/ml TNFα for 30 min, and then fixed. NF-κB p65 protein was visualized by indirect immunofluorescence staining using an antibody for p65. Arrows indicate mCherry-positive cells. The scale bar represents 50 μm. Bar graph shows quantitative analysis of NK-κB-translocated cells. Cells, in which nuclear NF-κB staining was visible as clearly as the merge of the nucleus was detectable, were regarded as NF-κB-translocated cells, and their percentage was calculated. 11–52 transfected cells were counted in each field. (*n* = 4). hBACCS2 enhanced cytosolic retention of NF-κB significantly in TNFα-cells. *p < .05 vs. hBACCS2 -, ^#^ *p* < .05, ^##^ *p* < .01 vs. blue light -, one-way ANOVA with Fisher’s LSD *post hoc* test. The effect of TNFα was always significant (*p* < .05); this is not indicated in the panel.

For photostimulation, the fluorescence microscopic system described below was used for fluorescence (∼.4 mW/mm^2^, for Figures 1, 2, 4, see below) and blue light LED (∼.4 mW/mm^2^, for Figures 3, 5, 6; LIU470A, ThorLabs, Newton, NJ, United States) were used.

### 2.4 Fluorescence Ca^2+^ and NO imaging

Cells, spread on poly-L-lysine-coated coverslips were incubated with 5 μM Fluo-8/AM in standard extracellular fluid (ECF), which contained: 140 mM NaCl, 5.4 mM KCl, 2 mM CaCl_2_, 1 mM MgCl_2_, 10 mM glucose, and 20 mM HEPES (pH 7.4 with NaOH), for 30 min at 37°C, followed by de-esterification of the dye for another 30 min at room temperature (22°C–25°C). The coverslips containing dye-loaded cells were held in a recording chamber placed on the stage of an inverted microscope (U-RFL-T mercury lamp, U-FBNA mirror unit, XL73, Olympus, Tokyo, Japan). Blue light (470–495 nm, 500 msec exposure, .2 Hz) for Fluo-8 excitation works by activating hBACCS2-mediated Ca^2+^ increase, and emitted light was culled with a 500–550 nm band pass filter. The fluorescence was detected with a ×20 objective lens (Olympus) and a CMOS camera (ORCA-Flash 2.8, Hamamatsu Photonics, Hamamatsu, Japan), using HCImage software 4.3.5 (Hamamatsu Photonics). Fluorescence intensities (ΔF) were normalized to the initial values (t = 0). Cells were not stained and incubated with ECF when G-CaMP7 was time-lapse monitored.

For NO imaging, cells were prepared as above and incubated with 5 μM DAF-FM/DA (Itoh et al., 2000) in standard ECF including 1 mM L-arginine, in the absence or presence of 1 mM N^G^-nitro-L-arginine methyl ester (L-NAME) for 30 min at 37°C, followed by de-esterification of the dye for another 30 min at room temperature. Thereafter, the procedure was the same as for Ca^2+^ imaging.

### 2.5 Reverse transcription and quantitative real-time PCR

Total RNAs of F2 cells were extracted with NucleoSpin RNA Plus (Takara). cDNAs were synthesized from 500 ng total RNA in 20 μl reactions using oligo (dT)_15_ and reverse transcriptase (Toyobo, Osaka, Japan).

Quantitative real-time PCR was performed to validate the expression levels of selected genes using SYBR^®^ Premix Ex Taq (Takara) and the Thermal Cycler Dice Real Time System (Takara) in accordance with the manufacturer’s protocols. The PCR amplification cycles consisted of denaturation at 95°C for 30 s, 40 cycles of denaturation at 95°C for 5 s, and annealing/extension at 60°C to for 60 s, followed by the detection of melt curve, 65°C–95°C. Real-time PCR reactions were carried out in duplicate for each sample, and the average values were applied to the ΔΔCt method for data analysis. Primer sets are described in “Supplementary Table S1”.

### 2.6 Microarray analysis

RNA samples (one from each condition) were used for global gene expression profiling. Microarray analysis was performed by Filgen Inc. (Nagoya, Japan) using the Clariom S array for mice (Thermo Fisher), for ∼22,000 genes, and GeneChip Scanner 3000 7G System (Thermo Fisher). The Microarray Data Analysis Tool version 3.2 (Filgen Inc.) was used for data normalization and subsequent processing. Differentially expressed mRNAs were identified using a set cutoff (signal intensities > 24, which is close to the median of negative control values, and fold change > 2 or < .5). Selected genes were processed for pathway analysis and those involved were classified based on gene ontology (GO) terms. A statistically ranked list of GO terms was then generated in terms of the z-score, which is a statistical measure of the relative amounts of gene expression changes in a given GO term (Doniger et al., 2003). A z-score of more than 3 is considered a statistically significant association between the differentially expressed genes and their corresponding GO terms. GO terms describing fewer than two genes that met the user-defined criteria were not considered in this study because of difficulty in ruling out coincidence.

Microarray data has been deposited in the Gene Expression Omnibus (GEO) at the National Center for Biotechnology Information (NCBI) (accession number GSE214156).

### 2.7 Reporter gene assays

Cells grown on 24-well plates were transfected with either pGL4.30[luc2P/NFAT-RE/Hygro] or pGL4.32[luc2P/NF-κB-RE/Hygro] (Promega) together with pRL-TK (Promega) to compensate for transfection efficiency. Expression plasmids were transfected in the following amounts per well: .2 μg of mCherry-containing vector, .15 μg of pGL4.30[luc2P/NFAT-RE/Hygro], .05 μg of pRL-TK for F2 cells. Luciferase assays were performed using the Dual-Luciferase Reporter System (Promega) with microplate luminometer (Molecular Devices, San Jose, CA, United States) according to the manufacturer’s instruction. For chelation of intracellular Ca^2+^, cells were pretreated with either BAPTA/AM (25 μM) or vehicle (dimethyl sulfoxide) for 30 min, after which blue light was applied.

### 2.8 Immunoblotting

Immunoblotting was performed as described (Inoue et al., 2010; Inoue et al., 2012). Cells cultured on 35 mm dishes were lysed in lysis buffer [50 mM Tris-HCl, pH 7.5, 100 mM NaCl, 1% Triton X-100, and protease inhibitor (Sigma-Aldrich)]. The lysates were collected after centrifugation at 14,000 × *g* and 4°C for 30 min. The aliquots were thereafter mixed with Laemmli sample buffer and boiled at 95°C for 10 min. The samples were resolved in 4%–20% SDS-PAGE, followed by electrotransfer to polyvinylidene difluoride membranes. For visualization, blots were probed with antibodies against Flag (1:1000) or GAPDH (1:3000), and detected using horseradish peroxidase-conjugated secondary antibodies (1:2000; Promega) and an ECL kit (Bio-Rad, Hercules, CA, United States).

### 2.9 Immunofluorescence staining

Cells were fixed with 4% paraformaldehyde in phosphate-buffered saline (PBS), followed by permeabilization in PBS containing .2% Triton X-100. The cells were first incubated with antibodies against Flag (1:1000) or NF-κB (1:500), and then with the Alexa 488-conjugated secondary antibodies (Thermo Fisher). For DNA staining, coverslips were incubated with 4′,6′-diamidino-2-phenylindole (DAPI). Fluorescent images were analyzed using a fluorescence microscopy (XL73, Olympus, Tokyo, Japan).

### 2.10 Statistical analysis

Data are presented as means ± SEM. Kaleidagraph 4.0 (Synergy Software, Reading, PA, United States) was used for statistical analysis. Differences between two groups were compared using an unpaired Student’s *t*-test. Comparisons among three or more groups were performed by one-way ANOVA, followed by Fisher’s least significant difference (LSD) *post hoc* test where applicable. *p* < .05 was regarded as statistically significant.

## 3 Results

### 3.1 Detection of Flag/hBACCS2-mediated G-CaMP activation by blue light irradiation

Numerous literature have been published on light-sensitive intracellular Ca^2+^ modulators (Fukuda et al., 2014; Ma et al., 2017a). Among them, as reported by Ishii et al. (2015) is a plasmid that bicistronically expresses mCherry and hBACCS2, which causes an intracellular Ca^2+^ increase when exposed to blue light. hBACCS2 is a fusion protein of phototropin 1, a light sensor, and STIM1, which binds to the Ca^2+^ channel ORAI1. Exposure to blue light irradiation facilitates a conformational change in the fusion protein, leading to binding and opening of ORAI1 and subsequent Ca^2+^ influx, sufficient for stimulation of downstream cellular processes (Ishii et al., 2015). To obtain enhanced expression levels in a variety of cells, we constructed a plasmid expressing Flag-tagged hBACCS2 and mCherry under a CAG promoter, which is often used for intensive and *in vivo* expression (Niwa et al., 1991; Araki et al., 1997). As shown in Figures 1A,B, a band with the expected molecular size (∼55 kDa) was detected using anti-Flag antibodies and the molecule was localized in the cytoplasmic region in HEK293 cells (Figures 1A,B). Blue light-induced Ca^2+^ increase was also observed in HEK293 cells containing the newly constructed Flag/hBACCS2-mCherry-expressing plasmid when cells were stained with a fluorescence Ca^2+^ indicator Fluo-8, while no change in fluorescence intensities were observed in either non-transfected or mCherry-positive (Flag/hBACCS2-negative) cells (Figure 1C).

For further convenience, we attempted to use G-CaMP7, an intracellularly expressed artificial Ca^2+^ sensor molecule, as opposed to staining with fluorescence indicators. G-CaMP7 is a derivative of GFP and its fluorescence intensity is Ca^2+^ concentration-dependent (Ohkura et al., 2012). Blue light application induced an elevation of fluorescence intensities in HEK293 cells expressing G-CaMP7 and Flag/hBACCS2-mCherry, whereas an elevation did not occur in the absence of Flag/hBACCS2 (Figure 1D). Removal of extracellular Ca^2+^ prevented increases in the fluorescence intensities, even in the presence of Flag/hBACCS2 (Figure 1D). These results suggest that photoactivation of Flag/hBACCS2 by blue light is detectable using G-CaMP7 fluorescence intensity. “Flag/hBACCS2” is hereafter simply represented as “hBACCS2”.

### 3.2 Ca^2+^ increase in the presence of hBACCS2 and blue light application in vascular endothelial cells

We examined whether the blue light/hBACCS2 system works in vascular endothelial cells, where the ORAI1-STIM1 system is present (Abdullaev et al., 2008; Li et al., 2011). In murine vascular endothelial F2 cells (Toda et al., 1990), fluorescence intensities of G-CaMP7 were elevated by blue light irradiation when hBACCS2 was present (Figures 2A,B). In contrast, hBACCS2-negative cells did not show a blue light-induced increase in G-CaMP7 fluorescence intensity. Under similar procedures, removal of extracellular Ca^2+^ prevented an increase in G-CaMP7 fluorescence intensities in hBCAACS2-positive cells (Figure 2B). An addition of EGTA, a Ca^2+^ chelator, also weakened the fluorescence (Figure 2B).

To obtain further insight into the availability of hBACCS2-dependent Ca^2+^ influx in vascular endothelial cells, we employed the b.End3 vascular endothelial cell line derived from brain vasculature. Similar to the case of F2 cells, fluorescence intensities of G-CaMP7 were elevated by blue light irradiation in hBACCS2-positive, but not hBACCS2-negative b.End3 cells (Figure 2C).

### 3.3 Intracellular Ca^2+^ signaling activation with hBACCS2 and blue light irradiation

Following observations of Ca^2+^ increase, we examined whether intracellular Ca^2+^-dependent signaling activity is influenced under our approach in vascular endothelial cells. Ishii et al. (2015) indicated that Ca^2+^-dependent activation of a transcription factor, nuclear factor of activated T cell (NFAT), was detected in HEK293 cells (Ishii et al., 2015). Consequently, we performed a reporter gene assay of NFAT in F2 cells. In this process, we challenged different conditions for 4 h in total, and cells that underwent 2 min/18 min cycles (repletion of 2 min irradiation and 18 min interval) showed the highest activity of NFAT reporter in the presence of hBACCS2 and blue light irradiation (Figure 3A). The photoactivation was eradicated when cells were pretreated with BAPTA/AM, a Ca^2+^ chelator (Figure 3B). Thus, a combination of blue light irradiation and presence of hBACCS2 can induce the activation of intracellular Ca^2+^ signaling.

### 3.4 NO production with hBACCS2 and blue light irradiation in vascular endothelial cells

NO production and release is an important function of vascular endothelial cells. Intracellular Ca^2+^ increase, such as by acetylcholine, activates eNOS, leading to enhanced NO production. We investigated whether NO production is facilitated by hBACCS2 and blue light irradiation. Transfected F2 cells were incubated with DAF-FM/DA, an NO-sensitive fluorescence indicator, and photostimulation was conducted. As shown in Figure 4, while hBACCS2-negative cells still displayed a slight increase in DAF-FM fluorescence intensities, hBACCS2-positive cells showed higher elevation (Figure 4). L-NAME, an NOS inhibitor, attenuated this increase (Figure 4). This indicated that photoactivation by the combination of blue light and hBACCS2 facilitates NO production.

### 3.5 Gene expression profiling of blue light-induced hBACCS2-positive vascular endothelial cells

Ca^2+^ signaling can also affect gene expression, as demonstrated by the altered activity of the transcription factor NFAT, as shown in Figure 3. To understand the global effect of Ca^2+^ elevation on gene expression in hBACCS2-transfected vascular endothelial cells, Clariom S microarray assay (Thermo Fisher) was used for analysis of RNA samples treated as in Figure 3 (2 min/18 min for 4 h). The scatter plot analysis in Figure 5A demonstrates that blue light irradiation resulted in up- and downregulated gene expression of 105 and 144 genes, respectively (Supplementary Tables S2, S3). To elucidate the possible molecular mechanisms associated with these differentially expressed genes, MAPP pathway analysis identified several pathways regulated by blue light irradiation, including apoptosis, myometrial relation/contraction, and inflammatory responses (Tables 1, 2). To validate the changes in gene expression of blue light-irradiated cells observed in microarray analysis, we performed quantitative real-time PCR for several target genes whose expressions were upregulated in hBACCS2-transfected F2 cells. As a result, blue light photoactivation upregulated the expression of genes indicated in Figure 5A in hBACCS2-positive cells [in contrast, the increase in ATF4 expression was non-significant (*p* = .058)], but not in hBACCS2-negative cells (Figure 5B). Although it was curious to us that the expression of E-selectin and ICAM-1, which are supposed to be expressed in a Ca^2+^-dependent manner, were not upregulated in this analysis (Chen et al., 2002), quantitative PCR also showed that Ca^2+^ increase alone is insufficient for its upregulation (Figure 5B, ICAM-1 was not detectable in our study, not shown). This may correspond with the results of a recent study that showed that extrinsic Ca^2+^ elevation by means of TRPV4 agonist does not increase E-selectin level (Chen et al., 2002; Beddek et al., 2021). Thus, consistent with microarray data, blue light mediated Ca^2+^ influx is possibly associated with vascular contractility and inflammation in the presence of hBACCS2.

**TABLE 1:** Up-regulated pathway by pathway analysis.

MAPP name	Number changed	Number measured	Z-score	*p*-value
Mm_Apoptosis_modulation_by_HSP70	2	17	6.67	.0037
Mm_Myometrialrelaxation_andcontraction_pathways	4	151	3.81	.0060
Mm_Mapk_signaling_pathway	4	158	3.69	.0070
Mm Oxidative stress response	2	27	5.16	.0084
Mm Inflammatory response_pathway	2	30	4.86	.0102
Mm Exercise-induced circadian_regulation	2	48	3.66	.0240
Mm_Lung fibrosis	2	61	3.13	.0368

**TABLE 2:** Down-regulated pathway by pathway analysis.

MAPP name	Number changed	Number measured	Z-score	*p*-value
Mm White fat cell differentiation	2	32	3.40	.0307
Mm Fatty acid beta-oxidation	2	33	3.34	.0324

### 3.6 Possible attenuation of NF-κB activity by blue light activation in hBACCS2-transfected vascular endothelial cells

Ca^2+^ elevation often evokes an attenuation of inflammatory responses in vascular endothelial cells (de Martin et al., 2000; Munaron, 2006; Bair et al., 2009; Li et al., 2017). In order to gain insight into this pathway during vascular inflammation, we examined whether inflammatory NF-κB activity is influenced by blue light and hBACCS2. It is well known that inflammatory mediators such as lipopolysaccharide and TNFα elicit vascular inflammatory activation. First, to inspect NF-κB activity, cells were treated with TNFα in the absence or presence of hBACCS2 and/or blue light irradiation, and a reporter system containing the NK-κB consensus sequence was challenged. While hBACCS2 simply enhanced basal reporter activities, TNFα induced NF-κB reporter activity in hBACCS2-transfected cells. The increased activity was significantly attenuated with blue light irradiation (Figure 6A). We then probed levels of nuclear translocation of NK-κB with TNFα treatment. Blue light-induced cytoplasmic retention of NF-κB was significantly greater in hBACCS2-positive cells than in hBACCS2-negative cells when stimulated with TNFα (Figure 6B). Taken together, although the functional effect of hBACCS2-dependent Ca^2+^ increase on TNFα-induced inflammatory responses was not significantly detectable, NF-κB translocation is likely to be influenced by the combination of hBACCS2 and blue light irradiation. This suggests that TNFα-induced inflammatory responses could possibly be attenuated with the combination of hBACCS2 and blue light with optimal photostimulatory doses.

## 4 Discussion

In blood vessels, vascular endothelial cells generate NO, an anti-coagulant that acts on platelets, and exhibit vasodilatory effects *via* vascular smooth muscles (Bauer and Sotnikova, 2010; Smolenski, 2012). The lack of NO production due to metabolic diseases such as hypertension and hypercholesterolemia can lead to atherosclerosis (Forstermann et al., 2017). Thus, regulation of NO production plays a critical role in vascular function. We attempted to manipulate cellular Ca^2+^ levels in cultured vascular endothelial cells, and consequently NO production and changes in gene expression by means of an optogenetic technique, which is widely available in neurobiological fields. Recently, studies using this technique for the cardiovascular system, have mainly focused on cardiac or vascular smooth muscle cells (Lee et al., 2021; Tong et al., 2021); to our knowledge, there is no literature focusing on vascular endothelial cells. As some optogenetic tools that can regulate Ca^2+^ levels are known, we employed a BACCS system which utilizes store-operated calcium entry, the ORAI1-STIM1 machinery, that is present endogenously in vascular endothelial cells. Among the currently used techniques that manipulate cellular function, the chemogenomic technique involves a designer receptor exclusively activated by designer drugs (DREADDs) which activates artificial G protein-coupled receptors through certain reagents (Urban and Roth, 2015; Aldrin-Kirk et al., 2016). However, the problems with DREADDs are: 1) introduction of artificial molecules into cells or tissues, 2) artificial drugs must be administered into the body, and 3) follow-up observation should be done, proving them harmless to the body (Keifer et al., 2020). Comparing the optogenetic approaches, some optogenetic tools also require introduction of artificial molecules into the body, and DREADDs may offer an advantage as their effects reach deeply in the body where light irradiation does not penetrate. Nonetheless, as optogenetics require less consideration of the impact of artificial drugs potentially involved in generation of the side effects, they can be considered more appropriate as a therapeutic strategy. This study initially aimed to examine whether our approach works in vascular endothelial cells; thus, we employed two vascular endothelial cell lines, F2 and b.End3 cells. As a result, we found that blue light irradiation induces an hBACCS2-dependent Ca^2+^ increase in both cells lines. Transfection efficiency and fold changes in fluorescence intensities of b.End3 cells were lower compared to those of F2 cells; therefore, we focused on F2 cells thereafter. Increase in intracellular Ca^2+^ levels induces calmodulin binding to eNOS, resulting in NOS enzymatic activity (Abu-Soud et al., 1994; McCabe et al., 2000). Consistent with this, we found that a combination of blue light and hBACCS2 induces Ca^2+^ influx and subsequent NO production. Thus, with incorporation of hBACCS2 into the vascular endothelium *in vivo*, it may be possible to control vascular function.

Apart from regulating cytoplasmic NO production, Ca^2+^ is regarded as a secondary messenger for intracellular signaling and plays a role in gene expression. NFAT activity, a known Ca^2+^-dependent factor, is also induced by the combination of hBACCS2 and blue light irradiation, and is eliminated in their absence. Moreover, intracellular chelation of Ca^2+^ with BAPTA eradicates NFAT activity, confirming that intracellular Ca^2+^ regulates NFAT transcriptional activity. As intracellular Ca^2+^ elevation is also known to alter gene expression *via* other transcription factors such as AP-1 (Salnikow et al., 2002; Liu et al., 2004), microarray analysis was carried out to depict a more comprehensive view of the cellular functions of Ca^2+^. The upregulation in adrenomedullin (ADM) expression, along with others, by hBACCS2/blue light was observed using microarray analysis in this study; however, the mechanism by which ADM responds to Ca^2+^ elevation was not pursued, along with potential AP-1 sites implicated in the human ADM promoter region (Ishimitsu et al., 2003), and this needs to be addressed for better understanding. ATF3 and ATF4 were also upregulated, and a recent study revealed that both of these genes expressed in vascular endothelial cells are critical for metabolic oxidative stress-induced angiogenesis (Fan et al., 2021), suggesting the possibility that optogenetic Ca^2+^ entry could support angiogenic potential. The benefit of this method of Ca^2+^ influx induction is that the expression of multiple beneficial molecules may be upregulated simultaneously. Although some detrimental genes could also be upregulated, it can be successful if an overall vascular or systemic improvement is observed.

Pathway analysis identified some pathways that are influenced by blue light irradiation. Regarding vascular endothelial roles, pathways for contractility and inflammatory responses were identified. Since inflammation dysregulates vascular contractility, resulting in vascular stiffness and atherosclerosis, we examined whether blue light irradiation affects inflammatory responses. In line with their relationship between arbitrary Ca^2+^ levels and inflammation, TNFα-induced NF-κB changes were monitored, and photostimulation of hBACCS2 attenuated its nuclear translocation and perhaps consequently its transcriptional activity. We provide a direction for future indications for the regulation of vascular function by arbitrary Ca^2+^ induction. Certain stimuli that potentially facilitate intracellular Ca^2+^ elevation can precondition vascular endothelial cells to detrimental stresses (Zahler et al., 2000; Jabs et al., 2010; Leonard et al., 2014), and the attenuation of inflammatory responses by Ca^2+^ increase in this study may induce this preconditioning. Surprisingly, arbitrary Ca^2+^ increase by blue light and hBACCS2 did not potentiate NF-κB, and thus the simple Ca^2+^ increase may not always be sufficient for NF-κB activation, as it sometimes needs other stimulation in addition to Ca^2+^ increase in other cells (Casolaro et al., 1995; Brignall et al., 2017). Of interest, as shown in Figure 6B, blue light irradiation alone may diminish the capacity of the inflammatory responses, as longer wavelengths have been indicated to have anti-inflammatory effects (Hamblin, 2017; Hamblin, 2018). Studies on optimization of light doses are required to move forward to *in vivo* studies.

Thus, although we found that hBACCS2 combined with blue light irradiation can activate vascular endothelial cellular functions, one of the limitations of this study is that we could not evaluate the optimal degree (e.g., intensity and frequency) of blue light stimulation. As performed by Ishii et al. (2015), we also performed an NFAT reporter assay under several conditions (data not shown) to obtain the optimum condition, and applied the most significant condition as found for microarray analysis. However, there might be more optimal conditions, that may result in greater improved functional changes. However, even if optimal stimulatory conditions are discovered in cultured cells, such stimulation may not always be transferrable *in vivo*; therefore, it would be better to start the search for optimal photostimulatory doses for *in vivo* studies. Also, in line with *in vivo* applications, a strong CAG promoter-containing expression plasmid was introduced in this study, which may cause vascular contraction when it is involved in neighboring vascular smooth muscle cells.

Ultimately, we hope to apply this method in a non-transgenic manner to treat human patients with conditions such as arteriosclerosis obliterans. However, testing the introduction of hBACCS2 into blood vessels by various methods in experimental animal models (Martin et al., 2000; Rutanen et al., 2002; Ouma et al., 2014; Young and Dean, 2015; Ma et al., 2017b; Goudy et al., 2019; Rasanen et al., 2021) should ideally precede any application in human. While introducing plasmid or viral vectors, it will be necessary to contain the effects of blue light in vascular endothelial cells, without affecting vascular smooth muscle cells (Wynne et al., 2009). Thus, this study presents the initial findings and a basis for further investigations on safe use of this approach *in vivo*. Further research is essential to allow this method to be promoted with *in vivo* applications in mind.

**FIGURE 7:**
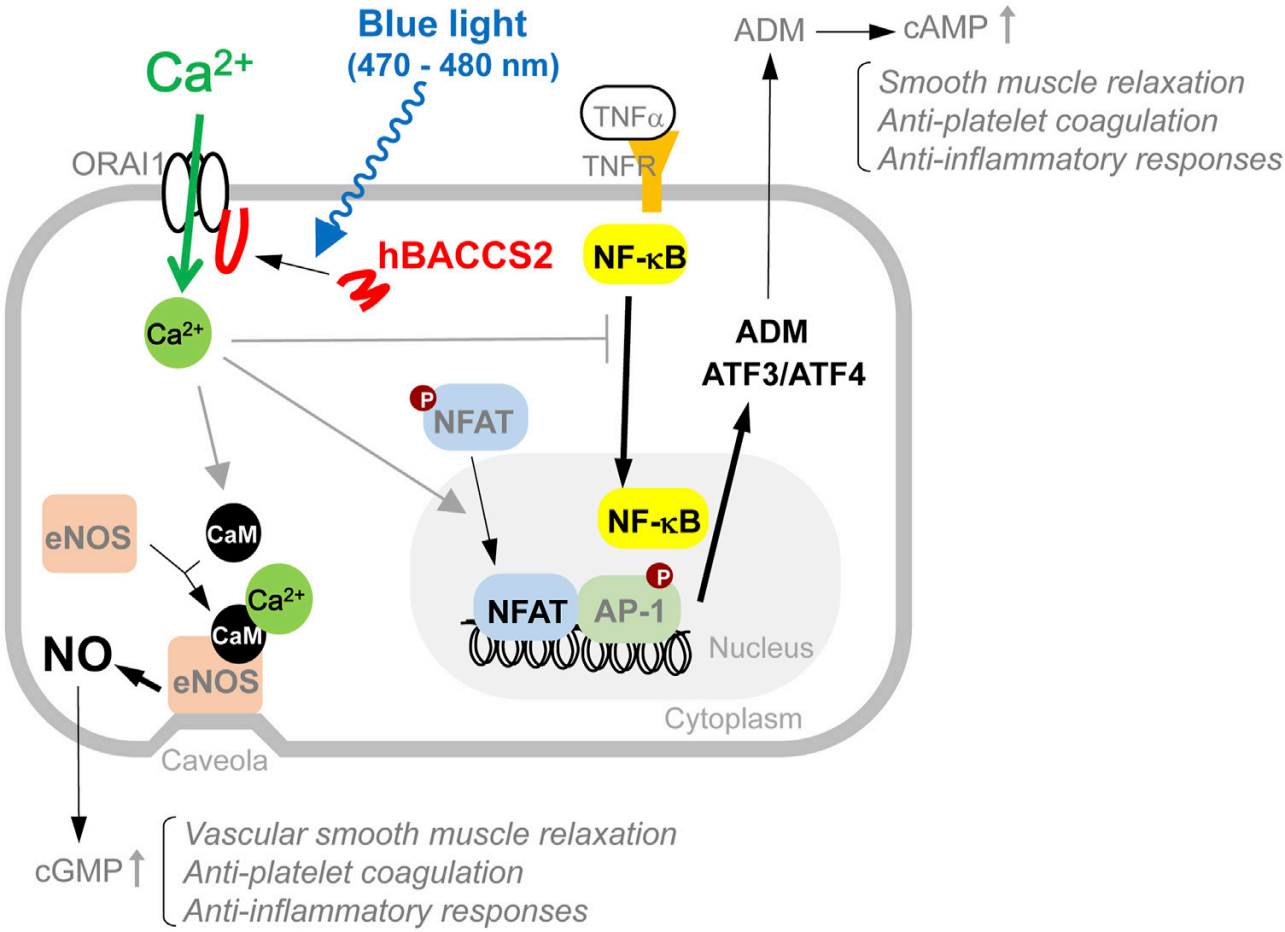
Model of intracellular events involved in blue light/hBACCS2-dependent Ca^2+^ regulation in vascular endothelial cells. Results from this study show that arbitrary increase in intracellular Ca^2+^ alters a variety of cellular events such as NO production and gene expression, resulting in changes in cellular function in vascular endothelial cells. Molecules observed in this study are indicated with bold and black letters, and the related changes are shown by thicker arrows.

A model of the arbitrary Ca^2+^-dependent cellular actions and their involvement in downstream possible anti-inflammatory signaling used in this study is depicted in Figure 7.

## 5 Conclusion

In this study, we employed a recently described optogenetic technique and found that blue light irradiation, along with hBACCS2 introduction, induces an arbitrary increase in Ca^2+^ in vascular endothelial cells, followed by a subsequent increase in intracellular Ca^2+^, NFAT activity and NO production. A comprehensive analysis of gene expression changes induced by blue light-dependent Ca^2+^ elevation using DNA microarrays revealed potential changes in the expression of genes related to smooth muscle contractility and inflammatory responses. The blue light-dependent Ca^2+^ increase also appeared to influence NF-κB activity induced by TNFα, an inflammatory mediator. These findings suggest that successful Ca^2+^ regulation of vascular endothelial cells may improve vascular function or regulate inflammatory responses. Further optimization of the stimulatory features and system, and its application *in vivo* are expected in the future.

## Data Availability

The datasets presented in this study can be found in online repositories. The names of the repository/repositories and accession number(s) can be found below: https://www.ncbi.nlm.nih.gov/, GSE214156.
