# Supplementary material for: Arbitrary Ca2+ regulation for endothelial nitric oxide, NFAT and NF-κB activities by an optogenetic approach
Source: Front Pharmacol. 2023 Jan 10;13:1076116. doi: 10.3389/fphar.2022.1076116 (PMC9871596; doi:10.3389/fphar.2022.1076116)
Supplement: Supplementary file 1 [file Table1.DOCX]

**Supplementary Information**

**Arbitrary Ca^2+^ regulation against vascular inflammation by an optogenetic approach**

Tomoyasu Yamanaka^1^, Takatoshi Ueki^2^, Mitsuhito Mase^1^, and Koichi Inoue^2^*

^1^Department of Neurosurgery, Nagoya City University Graduate School of Medical Sciences, Nagoya 467-8601, Japan

^2^Department of Integrative Anatomy, Nagoya City University Graduate School of Medical Sciences, Nagoya 467-8601, Japan

*** Correspondence:**Koichi Inoue
[ino-k@umin.ac.jp](mailto:ino-k@umin.ac.jp)

Phone: +81-52-853-8121

Fax: +81-52-853-8122

**Supplementary Table 1**

**Primers for real-time PCR**

| Gene | Forward(F)/Reverse(R) | Sequence |
| --- | --- | --- |
| GAPDH | F | 5’-TGCATCCTGCACCACCAACTGC-3’ |
|  | R | 5’-ACAGCCTTGGCAGCACCAGTGG-3’ |
| ATF3 | F | 5’-GGAAGAGCTGAGATTCGCCA-3’ |
|  | R | 5’-CTCATCTTCTTCAGGGGCCG-3’ |
| ATF4 | F | 5’-ATGGCCGGCTATGGATGATG-3’ |
|  | R | 5’-TCTGGCATGGTTTCCAGGTC-3’ |
| ADM | F | 5’-TTCACTCGCTTTCCTAGGCG-3’ |
|  | R | 5’-CACGACTTAGCGCCCACTTA-3’ |
| E-selectin | F | 5’-AGCAGAGTTTCACGTTGCAGG-3’ |
|  | R | 5’-TGGCGCAGATAAGGCTTCA-3’ |

**Supplementary Table 2**

**Up-regulated genes**

| Gene Accession | Gene Symbol | Gene Description | Ratio | Gene Accession |
| --- | --- | --- | --- | --- |
| NM_010478 | Hspa1b | heat shock protein 1B | 10.289177 | NM_010478 |
| NM_010479 | Hspa1a | heat shock protein 1A | 6.7314904 | NM_010479 |
| NM_010442 | Hmox1 | heme oxygenase 1 | 6.150793 | NM_010442 |
| NM_018808 | Dnajb1 | DnaJ (Hsp40) homolog, subfamily B, member 1 | 4.1639064 | NM_018808 |
| NM_026929 | Chac1 | ChaC, cation transport regulator 1 | 3.2806628 | NM_026929 |
| NM_178892 | Tiparp | TCDD-inducible poly(ADP-ribose) polymerase | 2.9934353 | NM_178892 |
| NM_026313 | Luc7l3 | LUC7-like 3 (S. cerevisiae) | 2.6530536 | NM_026313 |
| NM_025926 | Dnajb4 | DnaJ (Hsp40) homolog, subfamily B, member 4 | 2.6511397 | NM_025926 |
| NM_009174 | Siah2 | seven in absentia 2 | 2.61895 | NM_009174 |
| NM_145144 | Aif1l | allograft inflammatory factor 1-like | 2.5632307 | NM_145144 |
| NM_027859 | Rnf215 | ring finger protein 215 | 2.5511975 | NM_027859 |
| NM_007498 | Atf3 | activating transcription factor 3 | 2.5350801 | NM_007498 |
| NM_178799 | Acad12 | acyl-Coenzyme A dehydrogenase family, member 12 | 2.5315483 | NM_178799 |
| NM_001164671 | Dnaja1 | DnaJ (Hsp40) homolog, subfamily A, member 1 | 2.5121342 | NM_001164671 |
| NM_001013802 | Macrod2 | MACRO domain containing 2 | 2.5034941 | NM_001013802 |
| NM_134090 | Kdelr3 | KDEL (Lys-Asp-Glu-Leu) endoplasmic reticulum protein retention receptor 3 | 2.5031585 | NM_134090 |
| NM_012048 | Polk | polymerase (DNA directed), kappa | 2.5031144 | NM_012048 |
| NM_008528 | Blnk | B cell linker | 2.4918728 | NM_008528 |
| NM_001122738 | Kansl1l | KAT8 regulatory NSL complex subunit 1-like | 2.4870345 | NM_001122738 |
| NM_175275 | Cntln | centlein, centrosomal protein | 2.4838661 | NM_175275 |
| NM_001162872 | Exoc7 | exocyst complex component 7 | 2.4808659 | NM_001162872 |
| NM_172801 | Otop2 | otopetrin 2 | 2.4748228 | NM_172801 |
| NM_025844 | Chordc1 | cysteine and histidine-rich domain (CHORD)-containing, zinc-binding protein 1 | 2.467942 | NM_025844 |
| NM_013559 | Hsph1 | heat shock 105kDa/110kDa protein 1 | 2.4438706 | NM_013559 |
| NM_028756 | Slc35a5 | solute carrier family 35, member A5 | 2.4206625 | NM_028756 |
| NM_027041 | Cfap206 | cilia and flagella associated protein 206 | 2.4197584 | NM_027041 |
| NM_009226 | Snrpd1 | small nuclear ribonucleoprotein D1 | 2.3923947 | NM_009226 |
| NM_028500 | Calr3 | calreticulin 3 | 2.3909035 | NM_028500 |
| NM_001136059 | Cyp1a1 | cytochrome P450, family 1, subfamily a, polypeptide 1 | 2.3894518 | NM_001136059 |
| NM_026015 | Zmat5 | zinc finger, matrin type 5 | 2.3606371 | NM_026015 |
| NM_001110100 | Banp | BTG3 associated nuclear protein | 2.3574586 | NM_001110100 |
| NM_022331 | Herpud1 | homocysteine-inducible, endoplasmic reticulum stress-inducible, ubiquitin-like domain member 1 | 2.3545364 | NM_022331 |
| NM_001024919 | Bod1 | biorientation of chromosomes in cell division 1 | 2.3493719 | NM_001024919 |
| NM_001287180 | Atf4 | activating transcription factor 4 | 2.3492537 | NM_001287180 |
| NM_030016 | Trmt13 | tRNA methyltransferase 13 | 2.3488546 | NM_030016 |
| NM_001256100 | Mtif3 | mitochondrial translational initiation factor 3 | 2.3479935 | NM_001256100 |
| NM_027950 | Osgin1 | oxidative stress induced growth inhibitor 1 | 2.340965 | NM_027950 |
| NM_016710 | Hmgn5 | high-mobility group nucleosome binding domain 5 | 2.3283064 | NM_016710 |
| NM_009579 | Slc30a1 | solute carrier family 30 (zinc transporter), member 1 | 2.3133332 | NM_009579 |
| NM_178617 | Necab1 | N-terminal EF-hand calcium binding protein 1 | 2.3056682 | NM_178617 |
| NM_001082547 | Gm5483 | predicted gene 5483 | 2.2935631 | NM_001082547 |
| NM_027650 | Speer3 | spermatogenesis associated glutamate (E)-rich protein 3 | 2.2840819 | NM_027650 |
| NM_010765 | Mapkapk5 | MAP kinase-activated protein kinase 5 | 2.2747368 | NM_010765 |
| NM_177464 | R3hcc1l | R3H domain and coiled-coil containing 1 like | 2.2706536 | NM_177464 |
| NM_001034876 | Ccdc24 | coiled-coil domain containing 24 | 2.2702374 | NM_001034876 |
| NM_023168 | Grina | glutamate receptor, ionotropic, N-methyl D-aspartate-associated protein 1 (glutamate binding) | 2.2601879 | NM_023168 |
| NM_001130412 | Lpin1 | lipin 1 | 2.2363207 | NM_001130412 |
| NM_001033247 | Cfap44 | cilia and flagella associated protein 44 | 2.2343262 | NM_001033247 |
| NM_011484 | Stam | signal transducing adaptor molecule (SH3 domain and ITAM motif) 1 | 2.2179764 | NM_011484 |
| NM_176972 | Usp37 | ubiquitin specific peptidase 37 | 2.2171372 | NM_176972 |
| NM_174875 | Atg4a | autophagy related 4A, cysteine peptidase | 2.2116447 | NM_174875 |
| NM_009627 | Adm | adrenomedullin | 2.2009787 | NM_009627 |
| NM_001166743 | Vmn1r117 | vomeronasal 1 receptor 117 | 2.173639 | NM_001166743 |
| NM_013566 | Itgb7 | integrin beta 7 | 2.1730602 | NM_013566 |
| NM_028758 | Gga2 | golgi associated, gamma adaptin ear containing, ARF binding protein 2 | 2.1683045 | NM_028758 |
| NM_152804 | Plk2 | polo-like kinase 2 | 2.1622386 | NM_152804 |
| NM_011609 | Tnfrsf1a | tumor necrosis factor receptor superfamily, member 1a | 2.16217 | NM_011609 |
| NM_029788 | Rnft1 | ring finger protein, transmembrane 1 | 2.1589559 | NM_029788 |
| NM_001003393 | Igfl3 | IGF-like family member 3 | 2.1535236 | NM_001003393 |
| NM_001271388 | Ankra2 | ankyrin repeat, family A (RFXANK-like), 2 | 2.14949 | NM_001271388 |
| NM_026968 | Manbal | mannosidase, beta A, lysosomal-like | 2.1452553 | NM_026968 |
| NM_001242349 | Ano1 | anoctamin 1, calcium activated chloride channel | 2.141625 | NM_001242349 |
| NM_001271768 | Bhlhe41 | basic helix-loop-helix family, member e41 | 2.1370281 | NM_001271768 |
| NM_173028 | Vps13a | vacuolar protein sorting 13A (yeast) | 2.1335198 | NM_173028 |
| NM_023065 | Ifi30 | interferon gamma inducible protein 30 | 2.1322564 | NM_023065 |
| NM_011884 | Rngtt | RNA guanylyltransferase and 5-phosphatase | 2.1299144 | NM_011884 |
| NM_133435 | Nmnat1 | nicotinamide nucleotide adenylyltransferase 1 | 2.1288283 | NM_133435 |
| NM_028266 | Col16a1 | collagen, type XVI, alpha 1 | 2.1207461 | NM_028266 |
| NM_199465 | Nexn | nexilin | 2.119908 | NM_199465 |
| NM_001162903 | Ccdc71l | coiled-coil domain containing 71 like | 2.1143402 | NM_001162903 |
| NM_011173 | Pros1 | protein S (alpha) | 2.1128655 | NM_011173 |
| NM_025473 | Fam3a | family with sequence similarity 3, member A | 2.1079618 | NM_025473 |
| NM_001205099 | Npy2r | neuropeptide Y receptor Y2 | 2.1009171 | NM_001205099 |
| NM_026573 | Upf3b | UPF3 regulator of nonsense transcripts homolog B (yeast) | 2.0982956 | NM_026573 |
| NM_001037326 | Cstf3 | cleavage stimulation factor, 3 pre-RNA, subunit 3 | 2.0903904 | NM_001037326 |
| NM_177564 | Dhrs11 | dehydrogenase/reductase (SDR family) member 11 | 2.0884142 | NM_177564 |
| NM_053095 | Il24 | interleukin 24 | 2.0879868 | NM_053095 |
| NM_144804 | Depdc7 | DEP domain containing 7 | 2.0861573 | NM_144804 |
| NM_001081132 | Upf2 | UPF2 regulator of nonsense transcripts homolog (yeast) | 2.0823276 | NM_001081132 |
| NM_001009947 | Dock11 | dedicator of cytokinesis 11 | 2.0788974 | NM_001009947 |
| NM_001162489 | Mroh1 | maestro heat-like repeat family member 1 | 2.0768128 | NM_001162489 |
| NM_145620 | Rrp9 | RRP9, small subunit (SSU) processome component, homolog (yeast) | 2.0761507 | NM_145620 |
| NM_029078 | Pcf11 | cleavage and polyadenylation factor subunit homolog (S. cerevisiae) | 2.0758953 | NM_029078 |
| NM_026964 | Ccdc124 | coiled-coil domain containing 124 | 2.0754591 | NM_026964 |
| NM_001252639 | Tbc1d7 | TBC1 domain family, member 7 | 2.0751255 | NM_001252639 |
| NM_134218 | Vmn1r208 | vomeronasal 1 receptor 208 | 2.0663057 | NM_134218 |
| NM_001024731 | Gm20939 | predicted gene, 20939 | 2.0561046 | NM_001024731 |
| NM_177660 | Zbtb10 | zinc finger and BTB domain containing 10 | 2.0559543 | NM_177660 |
| NM_001142732 | Ttll3 | tubulin tyrosine ligase-like family, member 3 | 2.0492464 | NM_001142732 |
| NM_001167593 | Spata31d1b | spermatogenesis associated 31 subfamily D, member 1B | 2.0480477 | NM_001167593 |
| NM_001017429 | Cox17 | cytochrome c oxidase assembly protein 17 | 2.045 | NM_001017429 |
| NM_080448 | Srgap3 | SLIT-ROBO Rho GTPase activating protein 3 | 2.0406711 | NM_080448 |
| NM_013494 | Cpe | carboxypeptidase E | 2.0378559 | NM_013494 |
| NM_025312 | Sostdc1 | sclerostin domain containing 1 | 2.037182 | NM_025312 |
| NM_001166584 | Tead1 | TEA domain family member 1 | 2.0292479 | NM_001166584 |
| NM_008367 | Il2ra | interleukin 2 receptor, alpha chain | 2.0265069 | NM_008367 |
| NM_133662 | Ier3 | immediate early response 3 | 2.0173811 | NM_133662 |
| NM_175185 | Hsdl1 | hydroxysteroid dehydrogenase like 1 | 2.0158561 | NM_175185 |
| NM_134100 | Mfsd5 | major facilitator superfamily domain containing 5 | 2.0151484 | NM_134100 |
| NM_021790 | Cenpk | centromere protein K | 2.0148065 | NM_021790 |
| NM_008361 | Il1b | interleukin 1 beta | 2.0102767 | NM_008361 |
| NM_001205052 | Jdp2 | Jun dimerization protein 2 | 2.0093286 | NM_001205052 |
| NM_001111111 | Atg16l2 | autophagy related 16-like 2 (S. cerevisiae) | 2.0090424 | NM_001111111 |
| NM_011564 | Sry | sex determining region of Chr Y | 2.0058679 | NM_011564 |
| NM_029115 | Ccdc181 | coiled-coil domain containing 181 | 2.0055246 | NM_029115 |

**Supplementary Table 3**

**Down-regulated genes**

| Gene Symbol | Gene Description | Ratio | Gene Accession |
| --- | --- | --- | --- |
| Zfp953 | zinc finger protein 953 | 0.2564959 | NM_001038651 |
| Klf2 | Kruppel-like factor 2 (lung) | 0.2568816 | NM_008452 |
| Tbc1d8b | TBC1 domain family, member 8B | 0.2688369 | NM_001081499 |
| Fstl1 | follistatin-like 1 | 0.2781893 | NM_008047 |
| 2310009B15Rik | RIKEN cDNA 2310009B15 gene | 0.2791227 | NM_001081226 |
| Zfp39 | zinc finger protein 39 | 0.3260293 | NM_011758 |
| Ccnjl | cyclin J-like | 0.328827 | NM_001045530 |
| Zfp101 | zinc finger protein 101 | 0.337337 | NM_009542 |
| Pdgfb | platelet derived growth factor, B polypeptide | 0.3418894 | NM_011057 |
| Gstk1 | glutathione S-transferase kappa 1 | 0.342209 | NM_029555 |
| Ice2 | interactor of little elongation complex ELL subunit 2 | 0.3507903 | NM_145618 |
| Dxo | decapping exoribonuclease | 0.3515094 | NM_001163770 |
| Sox18 | SRY (sex determining region Y)-box 18 | 0.3524934 | NM_009236 |
| Prdm1 | PR domain containing 1, with ZNF domain | 0.3614714 | NM_007548 |
| Ch25h | cholesterol 25-hydroxylase | 0.363091 | NM_009890 |
| Trp53i11 | transformation related protein 53 inducible protein 11 | 0.3643826 | NM_001025246 |
| Lhx6 | LIM homeobox protein 6 | 0.3693194 | NM_001083125 |
| Zfp946 | zinc finger protein 946 | 0.3764109 | NM_198003 |
| Kdm6b | KDM1 lysine (K)-specific demethylase 6B | 0.3772502 | NM_001017426 |
| Zfp738 | zinc finger protein 738 | 0.3799857 | NM_001001187 |
| Sp2 | Sp2 transcription factor | 0.3803992 | NM_001080964 |
| Chst15 | carbohydrate (N-acetylgalactosamine 4-sulfate 6-O) sulfotransferase 15 | 0.3831598 | NM_029935 |
| Rhebl1 | Ras homolog enriched in brain like 1 | 0.3852234 | NM_026967 |
| Pxmp4 | peroxisomal membrane protein 4 | 0.3881347 | NM_021534 |
| Rgs2 | regulator of G-protein signaling 2 | 0.3901099 | NM_009061 |
| Id1 | inhibitor of DNA binding 1 | 0.392519 | NM_010495 |
| Myo1e | myosin IE | 0.3933861 | NM_181072 |
| Gbp2b | guanylate binding protein 2b | 0.3934849 | NM_010259 |
| Fzd4 | frizzled homolog 4 (Drosophila) | 0.3943776 | NM_008055 |
| Hinfp | histone H4 transcription factor | 0.3949443 | NM_172162 |
| Patz1 | POZ (BTB) and AT hook containing zinc finger 1 | 0.4019776 | NM_001253690 |
| Efna1 | ephrin A1 | 0.4032426 | NM_001162425 |
| 6330416G13Rik | RIKEN cDNA 6330416G13 gene | 0.4033392 | NM_144905 |
| Zfp945 | zinc finger protein 945 | 0.4078051 | NM_001110254 |
| Iqce | IQ motif containing E | 0.4081382 | NM_028833 |
| Pde4a | phosphodiesterase 4A, cAMP specific | 0.4083332 | NM_019798 |
| Pou2f1 | POU domain, class 2, transcription factor 1 | 0.4084577 | NM_011137 |
| Srgn | serglycin | 0.4091093 | NM_011157 |
| Tnfrsf8 | tumor necrosis factor receptor superfamily, member 8 | 0.4094447 | NM_009401 |
| Shroom3 | shroom family member 3 | 0.4120197 | NM_001077595 |
| Clp1 | CLP1, cleavage and polyadenylation factor I subunit | 0.4141969 | NM_133840 |
| Gemin8 | gem (nuclear organelle) associated protein 8 | 0.4151756 | NM_146238 |
| Lrch1 | leucine-rich repeats and calponin homology (CH) domain containing 1 | 0.4168501 | NM_001033439 |
| Mtch2 | mitochondrial carrier homolog 2 (C. elegans) | 0.4178244 | NM_019758 |
| Hipk2 | homeodomain interacting protein kinase 2 | 0.4197883 | NM_001136065 |
| Crybg3 | beta-gamma crystallin domain containing 3 | 0.4219122 | NM_174848 |
| Xrcc3 | X-ray repair complementing defective repair in Chinese hamster cells 3 | 0.4230734 | NM_028875 |
| Sox7 | SRY (sex determining region Y)-box 7 | 0.4234272 | NM_011446 |
| Nr3c1 | nuclear receptor subfamily 3, group C, member 1 | 0.4272195 | NM_008173 |
| Elovl6 | ELOVL family member 6, elongation of long chain fatty acids (yeast) | 0.4282535 | NM_130450 |
| Mat1a | methionine adenosyltransferase I, alpha | 0.4287291 | NM_133653 |
| Zfyve19 | zinc finger, FYVE domain containing 19 | 0.4327012 | NM_001164827 |
| Apeh | acylpeptide hydrolase | 0.4327741 | NM_146226 |
| Zfp217 | zinc finger protein 217 | 0.4335539 | NM_001033299 |
| Zfp366 | zinc finger protein 366 | 0.4339632 | NM_001004149 |
| Hpcal1 | hippocalcin-like 1 | 0.4339833 | NM_016677 |
| Cdk5rap1 | CDK5 regulatory subunit associated protein 1 | 0.4345178 | NM_025876 |
| Vmn1r7 | vomeronasal 1 receptor 7 | 0.4362691 | NM_001166710 |
| Nhsl1 | NHS-like 1 | 0.4373274 | NM_001163592 |
| Fbrsl1 | fibrosin-like 1 | 0.437397 | NM_001142642 |
| Zdhhc1 | zinc finger, DHHC domain containing 1 | 0.4376312 | NM_175160 |
| F2rl3 | coagulation factor II (thrombin) receptor-like 3 | 0.4415149 | NM_007975 |
| Gltscr1 | glioma tumor suppressor candidate region gene 1 | 0.4419331 | NM_001081418 |
| Prmt6 | protein arginine N-methyltransferase 6 | 0.4420642 | NM_178891 |
| Sap25 | sin3 associated polypeptide | 0.4454312 | NM_001081962 |
| Cc2d2a | coiled-coil and C2 domain containing 2A | 0.445798 | NM_172274 |
| C3ar1 | complement component 3a receptor 1 | 0.4468055 | NM_009779 |
| Dpy19l1 | dpy-19-like 1 (C. elegans) | 0.4479284 | NM_172920 |
| Tbx20 | T-box 20 | 0.4497861 | NM_001205085 |
| Birc3 | baculoviral IAP repeat-containing 3 | 0.4499091 | NM_007464 |
| Atg2a | autophagy related 2A | 0.450682 | NM_194348 |
| Speer4c | spermatogenesis associated glutamate (E)-rich protein 4C | 0.4510212 | NM_001281511 |
| Glyat | glycine-N-acyltransferase | 0.4510542 | NM_145935 |
| Zmiz1 | zinc finger, MIZ-type containing 1 | 0.4524072 | NM_183208 |
| 4930544D05Rik | RIKEN cDNA 4930544D05 gene | 0.4524336 | NM_001145537 |
| Pacs2 | phosphofurin acidic cluster sorting protein 2 | 0.4526854 | NM_001081170 |
| Cnksr3 | Cnksr family member 3 | 0.4544443 | NM_172546 |
| Ccdc14 | coiled-coil domain containing 14 | 0.4548496 | NM_172824 |
| Meis2 | Meis homeobox 2 | 0.4556727 | NM_001136072 |
| Epm2aip1 | EPM2A (laforin) interacting protein 1 | 0.4558061 | NM_175266 |
| Klf8 | Kruppel-like factor 8 | 0.4597078 | NM_173780 |
| Dnaja2 | DnaJ (Hsp40) homolog, subfamily A, member 2 | 0.4597085 | NM_019794 |
| Chkb | choline kinase beta | 0.4602714 | NM_007692 |
| Rexo1 | REX1, RNA exonuclease 1 homolog (S. cerevisiae) | 0.460519 | NM_025852 |
| Bdkrb2 | bradykinin receptor, beta 2 | 0.4607528 | NM_009747 |
| Kif13b | kinesin family member 13B | 0.4610473 | NM_001081177 |
| Dclk2 | doublecortin-like kinase 2 | 0.4617458 | NM_001195496 |
| Cand2 | cullin-associated and neddylation-dissociated 2 (putative) | 0.4621878 | NM_025958 |
| Metap1d | methionyl aminopeptidase type 1D (mitochondrial) | 0.4622173 | NM_025633 |
| Mkl1 | MKL (megakaryoblastic leukemia)/myocardin-like 1 | 0.4625209 | NM_001082536 |
| Rapgef6 | Rap guanine nucleotide exchange factor (GEF) 6 | 0.4626401 | NM_001252494 |
| Psd3 | pleckstrin and Sec7 domain containing 3 | 0.4626433 | NM_177698 |
| Klf10 | Kruppel-like factor 10 | 0.4628604 | NM_001289471 |
| Ttc37 | tetratricopeptide repeat domain 37 | 0.4631595 | NM_001081352 |
| Asxl1 | additional sex combs like 1 | 0.4641102 | NM_001039939 |
| Myo10 | myosin X | 0.4641862 | NM_019472 |
| Mfsd3 | major facilitator superfamily domain containing 3 | 0.4665052 | NM_027122 |
| Atp8a1 | ATPase, aminophospholipid transporter (APLT), class I, type 8A, member 1 | 0.4666289 | NM_001038999 |
| Ccdc28b | coiled coil domain containing 28B | 0.4669505 | NM_025455 |
| Jag1 | jagged 1 | 0.4681344 | NM_013822 |
| Ppp1r16b | protein phosphatase 1, regulatory (inhibitor) subunit 16B | 0.4684776 | NM_001159662 |
| Mrgpre | MAS-related GPR, member E | 0.4692549 | NM_175534 |
| Zkscan8 | zinc finger with KRAB and SCAN domains 8 | 0.4695802 | NM_001251833 |
| Napepld | N-acyl phosphatidylethanolamine phospholipase D | 0.4697047 | NM_178728 |
| Tnk2 | tyrosine kinase, non-receptor, 2 | 0.4713472 | NM_001110147 |
| Shroom4 | shroom family member 4 | 0.4714393 | NM_001040459 |
| Fgd5 | FYVE, RhoGEF and PH domain containing 5 | 0.4717544 | NM_172731 |
| Zmat3 | zinc finger matrin type 3 | 0.4723667 | NM_009517 |
| Scyl3 | SCY1-like 3 (S. cerevisiae) | 0.4728104 | NM_001286002 |
| Rnf111 | ring finger 111 | 0.4737211 | NM_033604 |
| 9430020K01Rik | RIKEN cDNA 9430020K01 gene | 0.4737735 | NM_001081963 |
| Serpinb3a | serine (or cysteine) peptidase inhibitor, clade B (ovalbumin), member 3A | 0.4737855 | NM_009126 |
| Prickle2 | prickle homolog 2 (Drosophila) | 0.4743327 | NM_001081146 |
| 2810021J22Rik | RIKEN cDNA 2810021J22 gene | 0.474396 | NM_172403 |
| Dll4 | delta-like 4 (Drosophila) | 0.477469 | NM_019454 |
| Dus3l | dihydrouridine synthase 3-like (S. cerevisiae) | 0.4778299 | NM_144858 |
| Mical1 | microtubule associated monooxygenase, calponin and LIM domain containing 1 | 0.4793637 | NM_001164433 |
| Jkamp | JNK1/MAPK8-associated membrane protein | 0.4811115 | NM_001205067 |
| BC055324 | cDNA sequence BC055324 | 0.481116 | NM_201364 |
| Mamld1 | mastermind-like domain containing 1 | 0.4821512 | NM_001081354 |
| Uprt | uracil phosphoribosyltransferase (FUR1) homolog (S. cerevisiae) | 0.4847603 | NM_001081189 |
| Cd55 | CD55 molecule, decay accelerating factor for complement | 0.4866285 | NM_010016 |
| Prr14 | proline rich 14 | 0.4870414 | NM_145589 |
| Acsf2 | acyl-CoA synthetase family member 2 | 0.4870848 | NM_153807 |
| Chst11 | carbohydrate sulfotransferase 11 | 0.4873982 | NM_021439 |
| Phf6 | PHD finger protein 6 | 0.4879346 | NM_027642 |
| Taf2 | TAF2 RNA polymerase II, TATA box binding protein (TBP)-associated factor | 0.4886105 | NM_001081288 |
| Dnajc30 | DnaJ (Hsp40) homolog, subfamily C, member 30 | 0.4889559 | NM_025362 |
| Gatsl2 | GATS protein-like 2 | 0.489431 | NM_030719 |
| Dennd5b | DENN/MADD domain containing 5B | 0.4908695 | NM_177192 |
| Gga3 | golgi associated, gamma adaptin ear containing, ARF binding protein 3 | 0.4909856 | NM_001252067 |
| Hspbap1 | Hspb associated protein 1 | 0.491152 | NM_175111 |
| Reep6 | receptor accessory protein 6 | 0.4913968 | NM_001204931 |
| Slc9a6 | solute carrier family 9 (sodium/hydrogen exchanger), member 6 | 0.4918557 | NM_172780 |
| Ino80d | INO80 complex subunit D | 0.4924986 | NM_001081436 |
| Slc41a1 | solute carrier family 41, member 1 | 0.4926594 | NM_173865 |
| Cabin1 | calcineurin binding protein 1 | 0.4935153 | NM_172549 |
| Foxp1 | forkhead box P1 | 0.4939722 | NM_001197321 |
| Rfx5 | regulatory factor X, 5 (influences HLA class II expression) | 0.495414 | NM_017395 |
| Cfap97 | cilia and flagella associated protein 97 | 0.4957697 | NM_025747 |
| Glrx | glutaredoxin | 0.496304 | NM_053108 |
| Acsl6 | acyl-CoA synthetase long-chain family member 6 | 0.4972123 | NM_001033597 |
| Gtf3c2 | general transcription factor IIIC, polypeptide 2, beta | 0.4972984 | NM_027901 |
| Trove2 | TROVE domain family, member 2 | 0.4974747 | NM_013835 |
